# Case Report: Whole exome sequencing identifies compound heterozygous variants in the *TRAPPC9* gene in a child with developmental delay

**DOI:** 10.3389/fgene.2024.1415194

**Published:** 2024-08-09

**Authors:** Bingxuan Yu, Jing Chen, Shuo Yang, He Wang, Yuanyuan Xiao, Shanling Liu

**Affiliations:** ^1^ Department of Medical Genetics/Prenatal Diagnostic Center, West China Second University Hospital, Sichuan University, Chengdu, China; ^2^ Key Laboratory of Birth Defects and Related Diseases of Women and Children (Sichuan University), Ministry of Education, Chengdu, China

**Keywords:** developmental delay, *TRAPPC9*, variants, whole exome sequencing, frame shift

## Abstract

**Background:**

Developmental delay in children under 5 years old, which occurs globally with an incidence of 10%–15%, is caused by multiple factors including genetics, prenatal conditions, perinatal complications, postnatal influences, social factors, and nutritional deficiencies. Gene variants such as *EFNB1*, *MECP2* and *TRAPPC9* play a significant role in protein deformation and downregulation of nuclear factor κB (NF-κB) activity.

**Methods:**

A 3-year-old girl, who exhibits poor gross motor skills, personal-social development, auditory language, hand-eye coordination, and visual performance, was diagnosed with global developmental delay. Trio whole exome sequencing was conducted to identify the genetic etiology of her condition. The identified genetic etiology was then validated through Sanger sequencing and quantitative polymerase chain reaction (qPCR).

**Results:**

Genetic analysis revealed that the patient had compound heterozygous variants in the *TRAPPC9* gene. These include a c.1928del frameshift variant inherited from the unaffected father and a deletion in exon 12 inherited from the unaffected mother. According to the American College of Medical Genetics (ACMG) guidelines, these variants were classified as “likely pathogenic”.

**Conclusion:**

The study revealed that compound heterozygous *TRAPPC9* gene variants cause developmental delay in a Chinese girl. These variants have been classified as having significant pathogenic effect according to the ACMG criteria, suggesting a recessive genetic pattern and highlighting the importance of prenatal testing for future offspring. Furthermore, our findings expand the genotype spectrum of the *TRAPPC9* gene, and provide more comprehensive information regarding genetic counseling for children experiencing developmental delay.

## 1 Introduction

Development delay (DD) includes developmental disabilities, failure and degeneration. The prevalence of DD in children under 5 years old has been reported to range from 10% to 15%, while the incidence of global developmental delay (GDD) ranges from 1% to 3% (Choo et al., 2019). The causes of DD in young children are multifactorial and result from a combination of prenatal factors (such as genetic disorders, cerebral dysgenesis, vascular occlusion, etc.), perinatal factors (such as preterm birth, perinatal asphyxia, bilirubin-induced neurological dysfunction, etc.), or postnatal events (such as meningitis, hypernatremia, hyponatremia, etc.), along with social, nutritional, and other factors (Choo et al., 2019). Several clinical studies have reported gene variants, including *EFNB1*, *MECP2*, *ATRX*, *NAA10*, *ANKRD11*, *ZNF*699, *TRAPPC9* and others associated with DD as significant causes of congenital growth retardation (Yan et al., 2019; Alvarez-Mora et al., 2021; Biela et al., 2022). Early intervention is essential for optimizing the developmental course of children with DD. If detected later, the opportunity for early intervention is lost, leading to adverse outcomes such as learning difficulties and behavioral problems. Therefore, it is important to refine the genetic spectrum of this disorder (Shannon and Anderson, 2008).

The gene we were concerned with, TRAFFICKING PROTEIN PARTICLE COMPLEX, SUBUNIT 9 (*TRAPPC9,* OMIM:611966)*,* is associated with DD. Johannes Krämer et al. reported that all of 43 cases of *TRAPPC9* variants, all showed intellectual disability (ID) and DD (Krämer et al., 2021). Other reports, including one consanguineous family in Pakistan and two consanguineous families in Iran, exhibited DD associated with *TRAPPC9* variants (Yousefipour et al., 2021; Asif et al., 2022). The process by which *TRAPPC9* genetic variants cause DD has not yet been elucidated, but it may be related to functional alterations in the transport protein particle complex subunit 9. This protein belongs to transporter particle II (TRAPPII) and plays an important role in Golgi vesicle tethering and Golgi transport in mammalian cells, thus influencing both cell development and individual development. At the same time, the clinical phenotype associated with *TRAPPC9* variants is linked to a decrease in NF-κB activation, which is a transcription factor that plays a critical role in activating genes associated with various cellular processes, including inflammation, immune response, cell proliferation, and apoptosis (Mbimba et al., 2018; Aslanger et al., 2022). Variants in *TRAPPC9* are also associated with autism spectrum disorder (ASD). A whole exome analysis of two siblings in Turkey reported that *TRAPPC9* variants are not only linked to ID, but also to ASD and repetitive physical behaviors, such as clapping behavior (Bolat et al., 2022). The same procedure was applied to two Thai siblings with ASD and ID, and *TRAPPC9* heterozygous variants were reported through whole exome sequencing (Hnoonual et al., 2019). The explanation of the mechanism mainly focuses on the deformation of related proteins and the downregulation of NF-κB activity (Wilton et al., 2020).

We reported a patient presented with GDD and other clinical symptoms Through WES, we identified novel compound heterozygous variants in the *TRAPPC9* gene, consisting of a heterozygous deletion of exon 12 and a frameshift variant (c.1928del, p.Y643Sfs*3). Our findings enhance the understanding of disease-causing variants in *TRAPPC9* and provides valuable information for molecular diagnostics.

## 2 Materials and methods

### 2.1 Patients and clinical data

**FIGURE 1:**
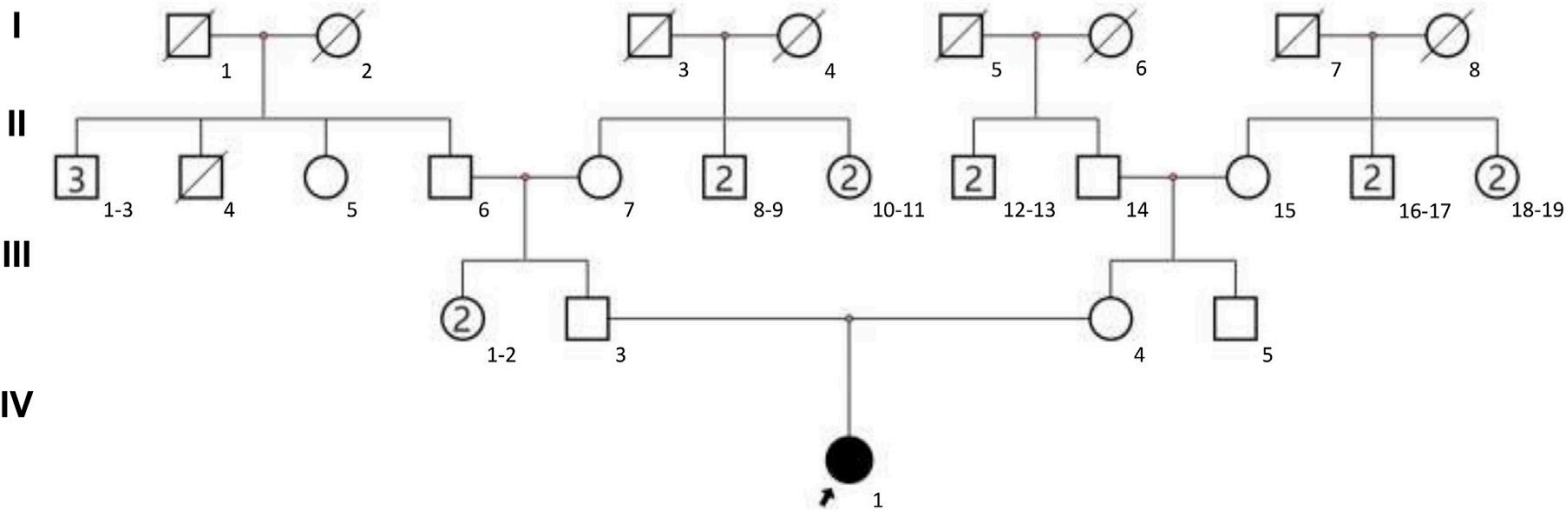
The family pedigree was depicted, with the proband designated by an arrow.

The present study focused on a 3-year-old female with GDD and other clinical symptoms from a Chinese family with no recorded family history. The family pedigree is illustrated in Figure 1. The child was admitted to West China Second Hospital at the age of three for medical evaluation and treatment. The findings of the brain magnetic resonance imaging (MRI) indicated abnormalities. The detailed clinical information of proband can be seen in Table 1. To investigate the genetic basis of the condition, Trio-WES was employed to analyze the family’s genetic profile. Subsequently, Sanger sequencing was used for validating variants initially detected through Trio-WES.

**TABLE 1:** The proband’s primary clinical symptoms.

Project	Clinical symptoms
Mode of delivery	A cesarean section was performed at full term
Developmental milestones	At 3 months: unable to head up
	At 8 months: can sit independently, but lacks stability
	At 1+ years old: unilateral facial convulsions and limb tremors occurred after the fall, lasting for approximately 10 min or more
	At 2+ years old: Can’t articulate words, such as ‘baba, mama’
	At the age of 2 and a half: start walking alone
Medical examination	The electroencephalogram is normal
	Brain MRI revealed abnormal signal in the lamellar of bilateral frontal cortex and white matter, as well as slight widening of bilateral lateral ventricle and third ventricle. The volume of white matter in the center of the semiovale was reduced, while the posterior part of the corpus callosum and splenium appeared thin. Additionally, there was widening and deepening of sulci and fissure
GESELL developmental assessment	Gross motor skills, personal-social skills, auditory language, hand-eye coordination, and visual expression are all behind those of children of the same age
Autism observation scale	Communication and social interactions: 16 points
	Game: 4 points
	Stereotypical behaviors and limited interests: 6 points
Anthropometric measurements (SDS*)	Height: 0.29
	Weight: 0.67
	Head circumference: −3.13
Current motor ability	Can walk alone, trot, but cannot maintain a steady pace
	Unable to jump Fine motor skills are basically normal
Language ability	Can only say two words of overlapping sounds
Special hobby	Like to run around aimlessly
Self-injury behavior	Hit the head repeatedly

*SDS, standard deviation score.

The written informed consent of all subjects has been obtained and approved by the Medical Ethics Committee of West China Second Hospital of Sichuan University.

### 2.2 DNA extraction

The genomic DNA of this family member was extracted from the blood, according to the manufacturer’s instructions (QIAamp DNA Blood Minikit). The proband and her parents’ genomic DNA was tested for WES, with sequences captured using Nano WES Human Exome V1 (Berry Genomics).

### 2.3 Whole exome sequencing

To identify variants in the proband, this study utilized the Nano WES Human Exome V1 for sequence capture and library formation. The enriched libraries were sequenced on the Illumina NovaSeq6000 platform, yielding 150 bp reads. The sequencing reads were then aligned to the hg38/GRCh38 reference genome using the Burrows-Wheeler Aligner (BWA) software (v0.7.17). Post-alignment, local recalibration of reads was conducted using the GATK Indel Realigner, and base recalibration was performed using the GATK Base Recalibrator (broadinstitute.org/). Single Nucleotide Variants (SNVs) and small indels were identified using the GATK Unified Genotyper. Variants with minor allele frequencies less than 0.05 were filtered using databases such as gnomAD (V3.1) (http://gnomad.broadinstitute.org/), the 1000 Genomes Project (phase 3) (http://browser.1000genomes.org). These selected variants were then subjected to pathogenicity prediction using platforms such as PolyPhen2 (http://genetics.bwh.harvard.edu/pph2), SIFT (http://sift.jcvi.org), and CADD (V1.6) (https://cadd.gs.washington.edu). Furthermore, to pinpoint causative variants, additional resources were consulted, including the National Institutes of Health, the Online Mendelian Inheritance in Man (OMIM,20230828) database (http://www.omim.org), the Human Gene Mutation Database (http://www.hgmd.org), and the ClinVar (20230819) database (http://www.ncbi.nlm.nih.gov/clinvar). The SNVs obtained in this way were divided into five categories, including pathogenic, likely pathogenic, uncertain significance, likely benign and benign, according to theACMG guidelines (Richards et al., 2015).

### 2.4 Sanger sequencing

To validate candidate variants, we sequenced PCR products of *TRAPPC9* c.1928del using an ABI 3500 genetic analyzer (Thermo Fisher Scientific) after amplifying each independent gDNA sample (both proband and her parents) with specific primer pairs 303 bp in length through polymerase chain reaction (PCR) amplification of each independent (Forward sequence: GAG​CAG​GAA​GCA​TGA​AGG; Reversed sequence: CTG​TCT​CCG​AAG​TTC​TCT​G).

### 2.5 Quantitative polymerase chain reaction (qPCR)

To validate the candidate variants selected by WES, qPCR was performed on gDNA from the proband and her parents using a SYBR Green qPCR master mix system and an Applied Biosystems 7500 real time PCR system. The copy number of *TRAPPC9* in each sample’s exon 12 was assessed using specific primer pairs through the 2 [-Delta Delta C (T)] analysis method (Livak and Schmittgen, 2001). Experiments were repeated three times for each sample. The forward sequence was TAG​ATT​TCC​AGT​GGG​TTC​AA, and the reversed sequence was TGT​GCC​TCA​GAC​ATT​AAG​TA.

## 3 Results

### 3.1 Compound heterozygous variants in *TRAPPC9* gene were found in a patients with developmental delay

**FIGURE 2:**
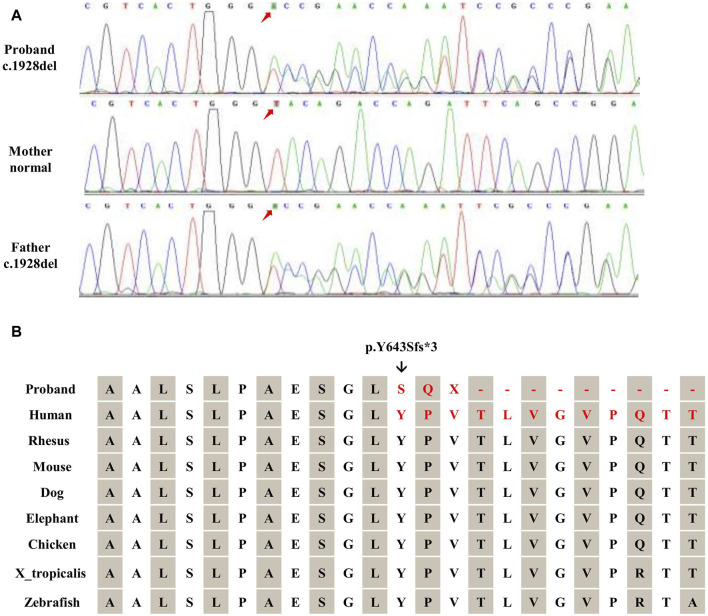
Validation of *TRAPPC9* SNVs. **(A)** Sanger sequencing was utilized to confirm the variant (c.1928del) within the family. **(B)** The frameshift mutation (c.1928del) in *TRAPPC9* leads to the substitution of glycine with a highly conserved arginine, resulting in a frameshift and premature termination (p.Y643Sfs*3).

**FIGURE 3:**
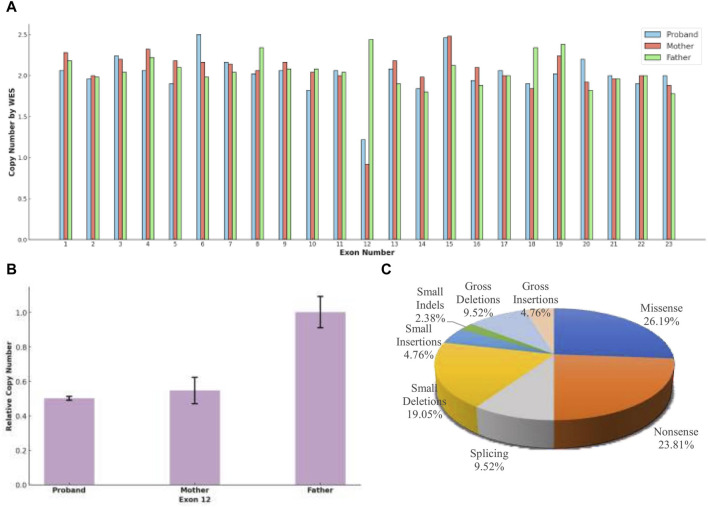
**(A)** Whole Exome Sequencing (WES) revealed that, except for exon 12, the copy number for all exons in the three subjects was approximately 2, indicating a normal range. Both the proband and the mother exhibited half of the normal copy number for exon 12, while the father’s exon 12 copy number was within the normal range. **(B)** A heterozygous deletion of *TRAPPC9* exon 12 was confirmed using qPCR. The analysis showed that the proband and the unaffected mother had half of the copy number for exon 12 compared to that of the unaffected father. **(C)** The distribution of disease-causing variation types in *TRAPPC9*, as cataloged in the Human Gene Mutation Database (HGMD^®^ Professional 2023.4), reflects the variety of genetic alterations associated with the gene.

The WES revealed a frameshift variant in *TRAPPC9* (NM 031466.8, c.1928del, p.Y643Sfs*3) from the paternal allele and a heterozygous deletion of *TRAPPC9* exon 12 (NM 031466.8) from the maternal allele. The frameshift variant was further validated by Sanger sequencing (Figure 2A) and the heterozygous deletion of exon 12 was confirmed by qPCR (Figure 3B). These results were consistent with WES, confirming the presence of this novel heterozygous compound variants.

The proband inherited a frameshift mutation derived from the paternal allele, resulting in a premature stop codon at position 645. This mutation was caused by the substitution of tyrosine with serine at the 643rd amino acid residue encoded by *TRAPPC9* exon 13 and the substitution of proline with glutamine at the 644th amino acid residue. Additionally, Figure 2B shows that multiple sequence alignment of the *TRAPPC9* gene from different species confirms the evolutionary conservation of amino acid residues at positions 643, 644, and 645 in this gene’s encoded protein. The proband also inherited a heterozygous deletion of exon 12 from their maternal allele, which was detected through WES analysis. qPCR experiments revealed that both the patient and mother had only half of normal copy number for *TRAPPC9* exon 12. However, this change was not found in the father (Figures 3A,B).

In summary, the proband’s clinical phenotypes were due to the compound heterozygous variants in the *TRAPPC9* gene, which included a maternally inherited heterozygous deletion of exon 12 in *TRAPPC9* and a paternally inherited frameshift variant c.1928del in *TRAPPC9*. According to the ACMG criteria, both compound heterozygous variants were classified as “likely pathogenic”.

## 4 Discussion

The *TRAPPC9* gene, also known as NIBP (NIK-and-IKK2-binding protein) or MRT13 (autosomal recessive mental retardation 13), plays a pivotal role in neurodevelopment (Abbasi et al., 2017; Hu et al., 2023). Animal studies have demonstrated that the defects in *TRAPPC9* leads to increased astrocyte proliferation in the hippocampus and corpus callosum of mice, which adversely affects the function of Rab11, a key protein involved in intracellular transport (Barrowman et al., 2010; Ke et al., 2020). *TRAPPC9* encodes for a protein believed to be involved in NF-kappa-B signaling (Hu et al., 2005). It acts as a subunit of the Transporter Particle Complex 9, which is a part of the TRAPPII complex. This complex is involved in various cellular processes, including protein secretion, autophagy, cytoplasmic division, and cilia formation (Barrowman et al., 2010). Clinically, variants in the *TRAPPC9* gene are closely linked to autosomal recessive intellectual disability (ARID) syndrome. This association is likely due to the gene’s role in regulating critical signaling pathways for brain development, particularly the NF-κB signaling pathway. Genetic studies have linked alterations in *TRAPPC9* to changes in brain structure and cognitive function, highlighting its significance in the study of neurodevelopmental biology and genetic disorders that result in intellectual disability (Bodnar et al., 2020). The involvement of this gene in such a wide range of critical developmental processes makes it a significant focus in neurogenetic research.

The *TRAPPC9* gene has been reported to have at least 42 variants classified as disease-causing mutations (DM), which encompass 11 missense variants, 10 nonsense variants, four splicing variants, eight small deletions, two small insertions, one small indels, four gross deletions, and two gross insertions in the HGMD database (Figure 3C). Among these variants, there are a total of 28 associated with ID, highlighting the significant role of the gene in cognitive development, and three variants have additionally been linked to DD. Boonsawat’s team reported on a patient with DD who had a *TRAPPC9* variant (c.3214C>T, p. Arg1072*), which led to altered neuronal NF-kappa-B signaling and was associated with moderate to severe GDD, mild cortical atrophy, and delayed myelination (Boonsawat et al., 2019). In another study, Masih’s team identified a patient with moderate DD with a *TRAPPC9* variant (c. 2458_2459delCT), and Koifman’s research group used chromosome microarray analysis and PCR technology to identify a 141.46 kb homozygous deletion in *TRAPPC9*, resulting in severe DD and agenesis of the corpus callosum (Koifman et al., 2010; Masih et al., 2022). The single exon deletion of *TRAPPC9* exon 12 in our case was initially identified through WES, which differs from the method employed by Koifman et al. WES tends to have high false positive rates in detecting single exon deletion due to uneven capture and less coverage of non-coding regions (Yao et al., 2017; O’Fallon et al., 2022). However, our result was confirmed by qPCR. The detection of this variant in the initial WES analysis may be attributed to the careful selection of control samples, enabling a comparison between control and test samples to identify differences and mitigate systematic errors. This emphasizes the importance of not overlooking copy number variants reported by WES in genetic analysis.

Our findings significantly contribute to the clinical management of the proband. One key insight is the potential link between the absence of *TRAPPC9* protein and an imbalance in dopamine D1 and D2 neurons, which might lead to impaired learning and memory (Ke et al., 2020). This suggests that drugs targeting D1 and D2 neurons could potentially alleviate these symptoms. Such a therapeutic approach could be particularly valuable in managing the neurological symptoms associated with *TRAPPC9* gene deficiency. Another innovative aspect involves using protein-protein interaction residues to investigate the binding properties of residues in both wild-type and mutant *TRAPPC9* proteins (Khattak and Mir, 2014). This approach has the potential to the development of drug therapies tailored to specific *TRAPPC9* variants, offering new avenues for therapeutic exploration in affected individuals. In our study, WES identified compound heterozygous variants in the *TRAPPC9* gene, including a single exon heterozygous deletion in exon 12 and a frameshift variant in exon 13, pinpointing the etiology of the global developmental of the proband. According to the ACMG criteria, both variants detected in *TRAPPC9* were classified as “likely pathogenic” and that parents who carries *TRAPPC9* gene variant, but exhibit no developmental delays, suggests a recessive genetic mode for the proband. This observation emphasizes the necessity of prenatal examination for subsequent offspring.

## 5 Conclusion

First, the observation of proband’s parents, each carrying one likely pathogenic variant of the *TRAPPC9* gene, exhibits no clinical symptoms, while the proband with compound heterozygous variants shows GDD, indicates a recessive inheritance pattern. The provision of this information is essential for genetic counseling and indicating that if the parents intend to have additional children, prenatal diagnosis should be offered to them. Additionally, our findings expand the genotypic spectrum of the *TRAPPC9* gene. Our findings provided valuable insights for the diagnosis and treatment of related disorders. The comprehensive understanding of the mutation spectrum in the gene can enhance the precision of genetic testing and inform the development of more effective therapeutic strategies for conditions associated with *TRAPPC9* variants.

## Data Availability

The variation data reported in this paper have been deposited in the Genome Variation Map (GVM) (https://ngdc.cncb.ac.cn/gvm/) in National Genomies Data Center, Beijing Institute of Genomics, Chinese Academy of Sciences and China National Center for Bioinformation, under accession number GVM000813.

## Abbreviations

DD, developmental delay; GDD, global developmental delay; WES, whole exome sequencing; qPCR, quantitative polymerase chain reaction; ACMG, American College of Medical Genetics and Genomics; *TRAPPC9*, TRAFFICKING PROTEIN PARTICLE COMPLEX, SUBUNIT 9; ID, intellectual disability; NF-κB, nuclear factor κB; ASD, autism spectrum disorder; NGS, next-generation sequencing; OMIM, Online Mendelian Inheritance in Man; SNVs, Single Nucleotide Variants; NIBP, NIK-and-IKK2-binding protein; ARID, autosomal recessive intellectual disability; MRT13, autosomal recessive mental retardation 13.
